# Locomo-Net: A Low -Complex Deep Learning Framework for sEMG-Based Hand Movement Recognition for Prosthetic Control

**DOI:** 10.1109/JTEHM.2020.3023898

**Published:** 2020-09-15

**Authors:** Arvind Gautam, Madhuri Panwar, Archana Wankhede, Sridhar P. Arjunan, Ganesh R. Naik, Amit Acharyya, Dinesh K. Kumar

**Affiliations:** 1 Indian Institute of Technology Hyderabad 233600 Hyderabad502205India; 2 RMIT University 5376 MelbourneVIC3001Australia; 3 Western Sydney University 6489 KingswoodNSW2747Australia

**Keywords:** sEMG, movement classification, signal processing, CNN, data compression, weights compression

## Abstract

**Background:** The enhancement in the performance of the myoelectric pattern recognition techniques based on deep learning algorithm possess computationally expensive and exhibit extensive memory behavior. Therefore, in this paper we report a deep learning framework named ‘Low-Complex Movement recognition-Net’ (LoCoMo-Net) built with convolution neural network (CNN) for recognition of wrist and finger flexion movements; grasping and functional movements; and force pattern from single channel surface electromyography (sEMG) recording. The network consists of a two-stage pipeline: 1) input data compression; 2) data-driven weight sharing. **Methods:** The proposed framework was validated on two different datasets- our own dataset (DS1) and publicly available NinaPro dataset (DS2) for 16 movements and 50 movements respectively. Further, we have prototyped the proposed *LoCoMo-Net* on Virtex-7 Xilinx field-programmable gate array (FPGA) platform and validated for 15 movements from DS1 to demonstrate its feasibility for real-time execution. **Results:** The effectiveness of the proposed *LoCoMo-Net* was verified by a comparative analysis against the benchmarked models using the same datasets wherein our proposed model outperformed Twin- Support Vector Machine (SVM) and existing CNN based model by an average classification accuracy of 8.5 % and 16.0 % respectively. In addition, hardware complexity analysis is done to reveal the advantages of the two-stage pipeline where approximately 27 %, 49 %, 50 %, 23 %, and 43 % savings achieved in lookup tables (LUT’s), registers, memory, power consumption and computational time respectively. **Conclusion:** The clinical significance of such sEMG based accurate and low-complex movement recognition system can be favorable for the potential improvement in quality of life of an amputated persons.

## I. Introduction

The myoelectric controlled powered prosthetic hands and limbs have the potential for improving the quality of life of amputated subjects [1]–[6]. The control of such prosthetic hands is facilitated by classifying various movements (e.g. finger and wrist) from the non-invasive recording of the muscular activity, known as surface electromyography (sEMG) [7], [8]. However, due to the poor quality of the acquired sEMG signals from the remnant muscles of the amputated limb, the real-time classification becomes very challenging [9]. To overcome this, there have been several approaches [10]–[14] reported thus far which can be broadly classified into the following four categories: (i) reduce the number of movements considered for classification, (ii) increase the number of body-worn electrodes, (iii) use of implantable electrodes and (iv) use of the multi-modal approach.

Surface electrodes have the advantage of being non-invasive and measure gross estimate of the muscle activity, but lack specificity. Reduction of the number of movements by the user improves the specificity of detection of the command from the signals [10]. However, this is at the cost of reduced dexterity and naturalness for the user, and thus user satisfaction. On the other hand, increasing the number of body-worn electrodes improves the performance of the classifier but the outcomes are dependent on the inconsistent placement of the electrodes making it unsuitable for self-administration [11].

The alternate is the implantable electrodes which have high specificity and are placed permanently by the surgeon, and thus do not require the user placement [12]. However, these necessitate a surgical procedure for implantation which limits the widespread usage of such devices. On the other hand, other approach like multi-modal where sEMG classifier’s outputs are combined with inertial measurement unit (IMU) and locational identifiers [13], [14] for better accuracy, but again their outcomes are limited to people performing specific activities in repetitive fashion. Thus, the desired system should be a myoelectric one with few electrodes and capability to identify several movements so that can provide the users convenience and natural dexterity.

In this context, many attempts have been made to improve the recognition of myoelectric based movements for the prosthetic hands [15]–[21]. These works can largely be categorized into two focal areas: (i) feature selection and (ii) classifier selection. The work by Soman *et al.* [15] used Twin-Support Vector Machine (SVM) for recognizing 15 wrist and finger flexion actions from the muscles of forearm using 4 channels of sEMG recording achieving average of 82% classification accuracy. Naik *et al.* [16] performed experiments to select the appropriate SVM kernel with 4 channels with similar accuracy (82.0%) but with only for 7 movements. Other studies have combined the classification parameters with feature selection [17]. Improvement in the results have been shown by using source separation using independent component analysis (ICA) [18]. These have demonstrated significantly improved accuracy of 95% with data collected from five transradial amputated participants. The traditional methodologies require supervision owing to its dependency on selection of suitable features from raw sEMG signal while achieving limited classification accuracy in the performance, thus restricts its usability for the real-time execution [22].

**FIGURE 1.**
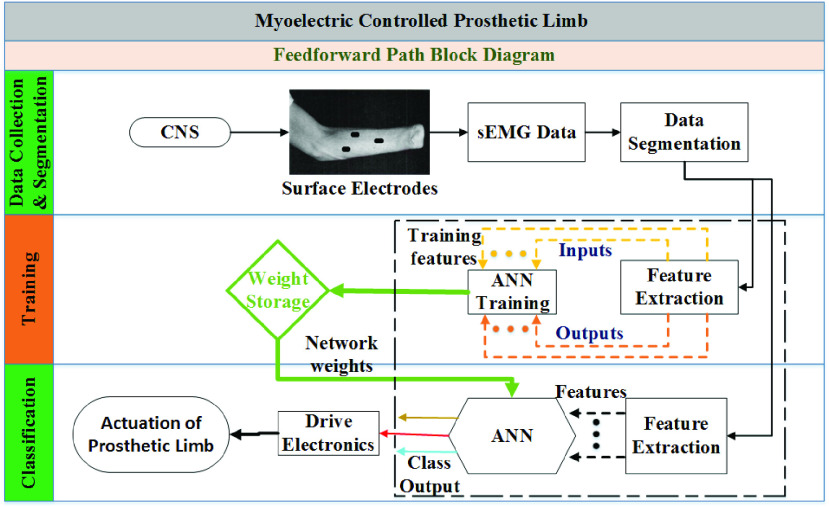
Shows the block diagram of the feed-forward path of a myoelectric controlled prosthetic limb which is bio-inspired and attempts to replicate the physiological motor control system [6]. There are a number of distinct tasks such as supervised data collection, segmentation, feature selection and extraction, training of the network followed by classification.

The deep-learning methods have the distinct advantage of performing the data-driven feature extraction while training, eliminates the computational time of feature selection [23]. Convolutional neural network (CNN) is one of the most widely used deep-learning approaches which performs data-driven feature extraction followed by classification (see Fig. 1) [22]–[26]. Recently, Park and Lee [19] introduced a CNN model for sEMG based hand movement classification and showed 90% accuracy for 6 movements. Later, Atzori *et al.* [9] employed CNN for classification of 50 movements in 67 intact subjects and 11 transradial subjects from Ninapro dataset (DS2) [27]. This method achieved an average accuracy of 55% while conventional classifiers such as linear discriminant analysis (LDA), SVM, k-nearest neighbor (k-NN) and Random Forest classifiers achieved 50%, 60%, 51% and 62% accuracy respectively. The recent study by Ulysse *et al.* [20], performed transfer learning using slow fusion model of CNN for hand gesture recognition from 8 channel sEMG recordings and reported an average accuracy of 97.8%. Another study by Zhai *et al.* [21], proposed CNN based self-recalibrating sEMG pattern recognition technique and obtained an average accuracy of 78.71% on NinaPro database for recognition of all movements. All of the above models used 3D spectrogram of sEMG as an input to CNN for recognition of different movements which adds extra computation cost for real-time implementation on resource constrained platforms. The CNN based pattern recognition techniques have automatic feature extraction capability which provides accurate recognition of the user movements from sEMG recordings. However, these are computationally expensive and exhibit extensive memory behavior for 3D input posing a bottleneck for its real-time implementation for a prosthetic limb where resources are scarce [28].

**FIGURE 2.**
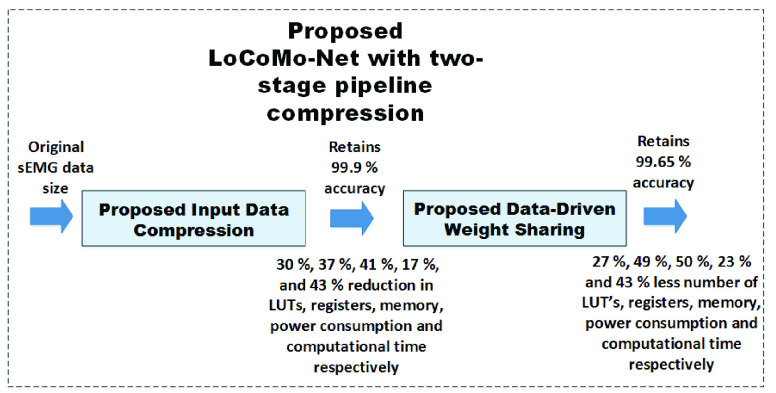
Proposed LoCoMo-Net with two-stage pipeline compression technique.

This paper reports the work to overcome the limitations of the earlier deep-learning methods, and develop a low-complexity CNN model without compromising the accuracy and the number of user commands. The proposed *LoCoMo-Net* framework is formulated for 1D data and includes two-stage pipeline: input data compression and a data-driven weight sharing, working holistically to reduce the computation and storage requirements as shown in Fig. 2. The two-stage pipeline architecture reduces complexity wherein stage 1 compresses the input by removing the redundant information and keeps the most informative data [29]. Next, in stage 2, the weights of the trained neural net model are compressed by indexing the neurons having the same weight. Further, to validate the feasibility of our proposed deep neural network for real-time execution, we have implemented on software-hardware co-designed solution for prosthetic control wherein average 27 %, 49 %, 50 %, 23 %, and 43 % savings achieved in lookup tables (LUT’s), registers, memory, power consumption and computational time respectively, depicting low-complexity nature of proposed deep neural network.

The remainder of the paper is structured as follows- Section II provides method for data acquisition system and the proposed *LoCoMo-Net* framework along with training and evaluation procedure, Section III presents the results, Section IV reports the discussion and Section V concludes the paper.

## II. Methods

### A. Data Acquisition System

#### 1) Dataset 1 (DS1)

The DS1 consists of eleven able-bodied (age: 26.6 ± 2.05 years, height: 170.6 ± 7.42 cm and weight: 70.6 ± 6.56 kg) and three trans-radial amputated participants (Characteristics are given in Table 6 in the appendix section). It was ascertained that the able-bodied volunteers did not present any evidence of skeletal, myo, or neuropathology diseases along with a normal range of motion without any restrictions. In this study, each individual was instructed to perform 15 tasks [15] excluding rest, which is listed in Table 7 in the appendix section −1.

**TABLE 1:** Neural Network Architectural Information of Different Models

Layer Type	NC Model		IC Model		LoCoMo-Net	
	O/P shape	Weights + biases	O/P shape	Weights + biases	O/P shape	Weights + biases
Input layer	250,1	0	146,1	0	146,1	0
Conv1D_1	246,7	42	142,7	42	142,7	42
Pooling_1	123,7	0	71,7	0	71,7	0
Dropout_1	123,7	0	71,7	0	71,7	0
Conv1D_2	119,7	252	67,7	252	67,7	161
Pooling_2	59,7	0	33,7	0	33,7	0
Dropout_2	59,7	0	33,7	0	33,7	0
Flatten	413	0	231	0	231	0
Dense (FC)	2	828	2	464	2	294

**TABLE 2:** Comparative Performance Analysis of NC (No-Compression), IC (Input-Compression) and LoCoMo-Net Models in Terms of Accuracy

Dataset		Movements	NC model Accuracy		IC model Accuracy		LoCoMo-Net model							
							Accuracy		Precision		Recall		F1-score	
			250 ms	200 ms	250 ms	200 ms	250 ms	200 ms	250 ms	200 ms	250 ms	200 ms	250 ms	200 Ms
DS1 Our Dataset	11 Able-bodied Subjects	16 Task movements including rest	93.5	92.9	93.5	92.7	93.4	92.5	93.8	93.4	98.2	98.0	95.9	95.6
	3 Amputated Subjects	16 Task movements including rest	89.2	88.5	89.1	88.1	88.8	87.9	92.3	91.9	93.6	93.1	92.9	92.5
DS2 Nina -Pro Dataset [27]	DB2- 40 Able-bodied Subjects	All movements (Index 1 to 50)	95.0	94.4	95.1	94.0	94.7	93.6	95.4	95.0	98.3	97.8	96.8	93.4
		Basics movement (Index 1 to 17)	96.5	95.8	96.4	95.5	96.2	95.3	96.9	96.6	99.2	98.9	98.0	97.7
		Grasping and functional movement (Index 18 to 40)	95.2	94.4	95.3	94.0	94.9	93.6	95.7	95.2	99.4	98.8	97.5	97.0
		Force pattern & rest (index 41 to 50)	92.1	91.8	92.2	91.4	91.8	90.8	92.2	91.8	94.1	93.5	93.1	92.6
	DB3- 11 Transradial Amputated subjects	All movements (Index 1 to 50)	89.0	88.1	88.9	87.9	88.8	87.6	91.7	92.1	93.9	93.4	92.8	92.8
		Basics movement (Index 1 to 17)	88.6	87.9	88.5	87.7	88.3	87.3	91.4	92.0	93.2	92.7	92.3	92.3
		Grasping and functional movement (Index 18 to 40)	89.7	88.6	89.6	88.4	89.4	88.0	92.3	92.7	94.6	94.0	93.4	93.3
		Force pattern & rest (index 41 to 50)	88.3	87.4	88.2	87.2	88.1	87.1	90.8	91.1	93.6	93.1	92.1	92.1

**TABLE 3:** Hardware Complexity Analysis for NC, IC and LoCoMo-Net Model for 15 Task Classification. (Memory Used Unit is Bit.)

Parameters			NC model	Proposed IC model		ProposedLoCoMo-Net model	
				CNN Model	Min/max technique	CNN model	Min/max technique
Total no. of LUT			5477	3781	55	3951	55
Total no. of Register			8800	5465	76	4405	76
DSP			4	4	–	4	–
BRAM	Global memory	Input	120K bit	60K bit	–	60K bit	–
		Conv.	63K bit	63K bit	–	50K bit	–
		Dense	180K bit	90K bit	–	58K bit	–
		Index	–	–	–	15K bit	–
	Local memory		13K bit	7K bit	–	7K bit	–
	Total		376K bit	220K bit	–	190K bit	–
On chip power consumption			178 mW	146 mW	2 mW	135 mW	2 mW
Computational time (worst case)			8.2 ms ($547.4~\mu \text{s}$/classifier)	$317~\mu \text{s}$/classifier	$5~\mu \text{s}$	$317~\mu \text{s}$/classifier	$5~\mu \text{s}$
				4.7 ms		4.7 ms

**TABLE 4:** Comparison of Classification Accuracy With State-of-the-Art Model

Movement	Twin-SVM [15] 250ms	LoCoMo-Net 250ms
Task 1	95	93.2
Task 2	84.5	92.5
Task 3	80.2	91.1
Task 4	81.6	85.2 91.2
Task 5	88.0	
Task 6	82.7	93.4
Task 7	83.6	93.2
Task 8	80.6	91.4
Task 9	83.3	92.2
Task 10	81.8	87.2
Task 11	75.8	92.3
Task 12	78.2	90.2
Task 13	80.2	88.2
Task 14	79.4	92.4
Task 15	83.7	93.5
Average	82.6	91.1

**TABLE 5:** Comparison of LoCoMo-Net Model Parameters With the State-of-the-Art Cnn Based Models (Where LC Represents Locally Connected Layer)

Parameter	[19]	[9]	[21]	[42]	[43]	LoCoMo-Net	[20]
Input length (ms)	–	150	200	200	200	200	260
Input Conversion penalty	Yes	Yes	Yes	Yes	Yes	NA	Yes
CNN Input	3D	3D	3D	3D	3D	1D	3D
Filter size	2D	2D	2D	2D	2D	1D	2D
Filter length	128	192	–	256	128	14	72
Conv layers	4	5	1	2	2	2	5
Pool layers	4	2	–	–	1	2	–
FC layers	2	–	2	3	2	1	2
LC layers	–	–	–	2	–	–	–
LSTM	–	–	–	1	–	–	–
Dropout	0.5	–	0.5	0.5	0.20	0.5	0.65
Dataset	DS2	DS2	DS2	DS2	DS2	DS2	Own
Movements	6	50	50	50	50	50	7
subjects	27	40	40	40	40	40	35
Average Accuracy	90.0	60.2	78.7	82.2	71.6	93.6	97.8

**TABLE 6:** Characteristics of the Trans-Radial Amputated Participants

Age	Sex	Amputated Hand	Stump Length (cm)	Stump Circ. (cm)	Time since Amputation (yrs.)	Use of Prosthe-sis
35	M	Right	26	24.5	3	Yes
60	F	Left	31	26	40	Yes
42	F	Right	24	25	35	Yes

**TABLE 7:** Task Performed During sEMG Recording

Serial No.	Task Description
Task 1	Movement of the little finger (Flexion)
Task 2	Movement of the ring finger(Flexion)
Task 3	Movement of the middle finger(Flexion)
Task 4	Movement of the index finger(Flexion)
Task 5	Movement of the thumb finger(Flexion)
Task 6	The combined movement of the little and ring fingers (Flexion)
Task 7	The combined movement of the ring and middle fingers together (Flexion)
Task 8	The combined movement of the little and index fingers together
Task 9	The combined movement of the index and middle fingers together
Task 10	Wrist flexion
Task 11	Wrist extension
Task 12	Wrist flexion and extension
Task 13	The combined movement of all fingers(Flexion)
Task 14	Hand grasp
Task 15	Pinch grip
Task 16	Rest

The experimental protocol was approved by the RMIT University Human Research Ethics Committee (Melbourne, Australia) and UFES, Vittoria, Brazil. All experiments were conducted in accordance with the Helsinki Declaration (revised 2004), and participants provided their oral and written consent before the start of the experiment. The experimental protocol was the same as used in our previous study [16]. For data collection, we used four bi-polar, active electrode sEMG system by Touchbionics Ltd, UK. The surface of the skin was cleaned with alcohol before the attachment of electrodes on the muscle surface. Then, a conductive electrolyte gel was applied to the electrodes, and the ground electrode was placed on the volar aspect of the wrist. The data were recorded using a LabVIEW based sEMG acquisition system with a sampling frequency of 1000 Hz for each channel at a resolution of 16 bits/sample.

**FIGURE 3.**
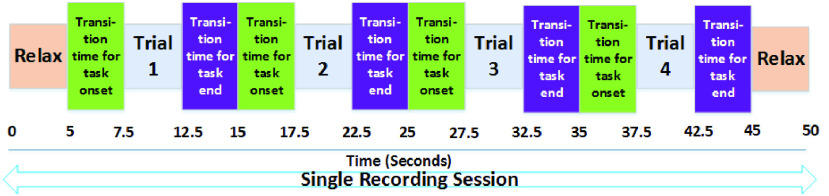
Experimental protocol for recording sEMG data. Where data was recorded in 4 trails for each activity discriobed in Table 2.

The placement of the bipolar electrode on the surface of the forearm is as follows- channel 1 is positioned at the Flexor pollicis longus muscle, channel 2 at the flexor digitorum superficialis muscle, channel 3 at the flexor carpi radial and ulnaris muscle, and channel 4 at the extensor carpi radial and ulnaris muscle. The participants were familiarized with the procedure, equipment, and demonstrated the task before starting the experiments. For this study, each subject was instructed to perform 15 tasks, excluding rest, which is listed in Table 7. Each task described in the Table 7 was performed in two sessions for the able-bodied participants which lasted for 50 seconds for single session as shown in Fig. 3. While, for the amputated participants only one session was performed to minimize the fatigue due to the experiment. Fig. 3 shows that the initial 5 seconds of the single session was allocated for the relaxation of the participants to get comfortable with the specific task followed by 4 trails of task activity of 10 seconds each which includes transition time for task onset and transition time for task end. The repeated movements from the trials are separated based on the time recorded in one of the channels when one repetition is completed. The participants repeated one task for 40 times in a session, which resulted in 80 sample points for a single task (referred to as Class) for able-bodied participants and 40 for trans-radial amputated participants.

#### 2) Dataset 2 (DS2)

In this study, the publicly available NinaPro database which has been reported in many studies for sEMG pattern recognition [9], [19], [21] was used as the second dataset named as DS2. The DS2 comprises of NinaPro database 2 (DB2) and database 3 (DB3) [27], consisting of 40 intact subjects, and 11 trans-radial amputated subjects respectively. The disabilities of the arm, shoulder, and hand (DASH) scores of the amputated subject was in the range of 1.67 to 86.67 (scale 0–100). Each subject was requested to perform 49 types of hand movements including 8 isometric and isotonic hand configurations; 9 basic wrist movements; 23 grasping and functional movements; and 9 force patterns. Each movement was repeated 6 times with a 3 s rest in between. The 12-channels sEMG signal was sampled at 2000 Hz. In this study, total of 50 movements (49 movements and 1 rest) were considered from Ninapro database.These include 17 basic movements of the finger and wrist (index 1 to 17); 23 grasping and functional movements (index 18 to 40); 9 force pattern (index 41 to 49) and rest (index 50) for comparison with the state-of-the-art CNN based models.

### B. LoCoMo-Net- Proposed Deep Learning Framework

**FIGURE 4.**
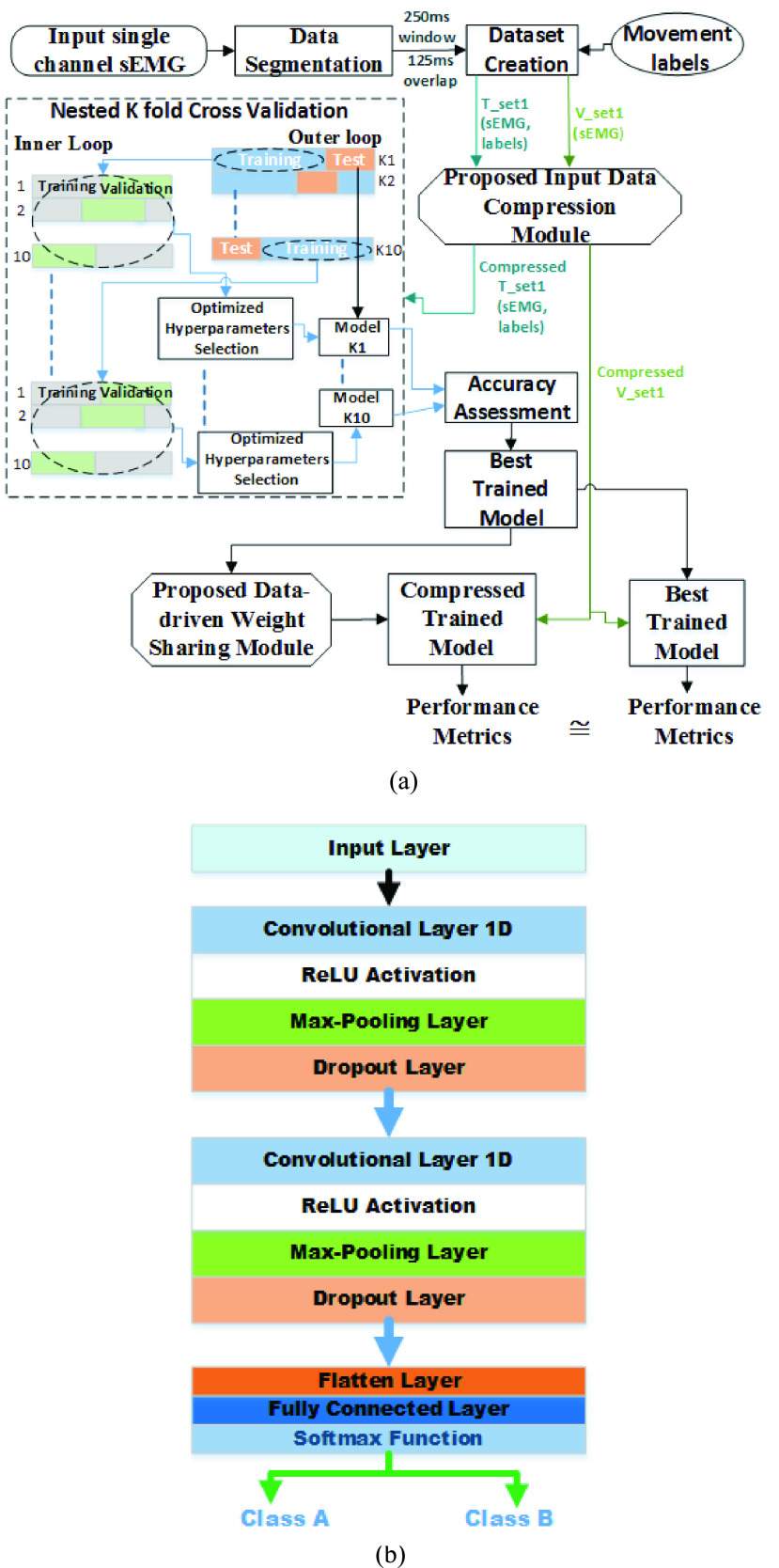
(a) Workflow of the proposed *LoCoMo-Net* where T_set1 and V_set1 represent the train-test and validating set of DS1 respectively. The Nested 10 fold CV is used for the selection of best hyper-parameters. (b) The network topology of the proposed *LoCoMo-Net* model for classifying the task.

Fig. 4(a) represents the workflow of the proposed deep learning framework *LoCoMo-Net,* consisting the CNN model with a two-stage pipeline: input data compression and a data-driven weight sharing. The *LoCoMo-Net* framework was formulated as one versus all (binary) classification problem due to its feasibility for training initialization as well as usage in a practical scenario [30].

For the proposed framework single channel (i.e. channel 1 of DS1) sEMG data was utilized for classification of movements with the aim to reduce the computation and complexity for real-time practice. While a matching channel (i.e. channel 1) was used for NinaPro dataset (DS2). The signal was segmented with 250 ms and 200 ms window with 50% overlap for DS1 and DS2 based on the literature to ensure the delay was within acceptable limits [31]–[33]. The recent study by [9] done the analysis on effect of normalization on the classification accuracy. The ‘time window normalization’ of data affects the accuracy most when compared to ‘normalization based on training data’ and ‘no normalization’.

Therefore, in our study sEMG data was normalized to zero mean and unit variance from the training dataset to avoid over fitting problem and faster conversion of gradient descent algorithm [34].

### C. Network Topology of the Proposed LoCoMo-Net Model

**FIGURE 5.**
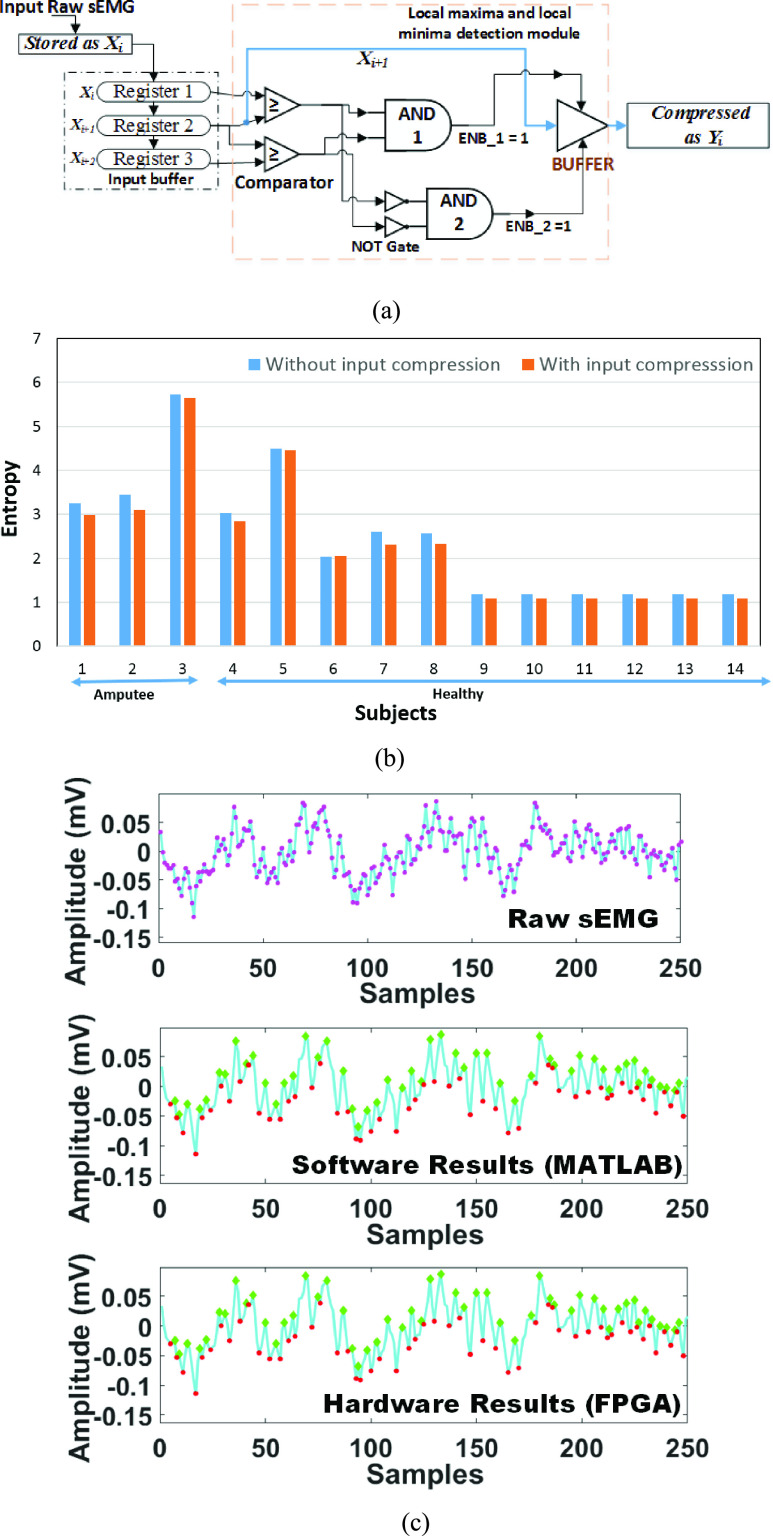
(a) Shows block level architecture of the proposed Min/max technique; (b) Mean entropy comparison of sEMG with and without input data compression for 3 amputated and 11 healthy subjects; (c) Comparison of the raw sEMG signal with compressed sEMG from the proposed input data compression module implemented on software (MATLAB) and hardware (FPGA), the analysis shows both the hardware and software results retains same morphology like raw sEMG with less number of samples.

The data-driven feature extraction for discriminatively classifying the subtle forearm movements from single channel sEMG recording was realized by using two 1D CNN layers, each followed by Rectified Linear Unit (ReLU) activation function, max-pooling layer, and dropout layer. After this, flatten layer was included to transform the 2D feature maps into 1D data. Lastly, a fully connected layer with softmax loss function was used for the classification of a particular task based on the features extracted from the previous layers. This entire network topology of the proposed *LoCoMo-Net* is depicted in Fig. 4(b). In this, softmax loss function outputs the probabilities of the different classes considered for the classification and then performs the classification based on the cost function from the normalized exponential function. Since, we have formulated this particular problem as a binary classification, therefore each task has two classes: class A and class B where class A is the *‘specified task’* and class B is the ‘ *other*’. The proposed model was formulated after exploring the hyper-parameter tuning [35] with different hyper-parameters where 2 convolutional layers, 7 filters of size $5\times 1$, stride rate of 1 in both convolutional layers and $2\times 1$ max-pooling were chosen as optimum parameters for the given problem. This resultant in 213 neurons in the fully connected layer for *LoCoMo-Net* which is also illustrated in Fig 5. For training the model, categorical cross-entropy loss function, RMSprop optimizer with a default learning rate of 0.001 and the batch size of 15 are incorporated where maximum 50 epochs are used. Dropout is included in the training to overcome the over-fitting issue on new unseen data which is set to recommended 50% probability level. To generalize the proposed framework, data of all the subjects were included during training for each task.

### D. Evaluation Methodology

The sEMG signal is non-stationary and sensitive to many factors, such as electrode placement, signal crosstalk and recording environment [41]. As a result, it leads to a significant variation in the sEMG data captured from the same subject muscles for the same activity performed in different trials. Therefore, for performance evaluation of the *LoCoMo-Net*, each dataset (DS1 and DS2) was divided into two parts train-test set and validate set. To formulate the model for each movement in DS1, Trial 1, Trial 3 and Trial 4 from all the subjects were included in a train-test set named as T_set1, whereas Trial 2 was used as validating set named as V_set1. While in DS2, $1^{\mathrm {st}}$, $3^{\mathrm {rd}}$, $4^{\mathrm {th}}$, and $6^{\mathrm {th}}$ numbered repetitions from all the subjects were considered as a train-test set (T_set2), whereas $2^{\mathrm {nd}}$ and $5^{\mathrm {th}}$ repetition was used as validating set (V_set2) exactly the same way as reported in [9], [21].

To get the optimum model for the given application, hyper-parametrization was performed with T_set1 of DS1, wherein nested k-fold cross-validation method was adopted to get the unbiased evaluation for the proposed model from the literature [39] for k equal to 10. The nested k-fold validation ensures that no information is leaked to the model, thereby providing an unbiased evaluation which was done by utilizing the Gridsearch cross validation [40] in python. To check the stability of the selected model, the same parameters from the DS1 are used for evaluation of the DS2 dataset. For DS2, T_set2 was used to train the model with parameters obtained from DS1. After training the model, it was validated with the unseen V_set2, and then results are tabulated. The efficiency of the proposed *LoCoMo-Net* is measured in terms of accuracy, precision, recall, F1-score, receiver operating characteristics (ROC) analysis, computation time, power-area analysis and resource utilization. Subsequently, a comparative analysis was also performed with state-of-the-art models using the DS1 and DS2 datasets.

### E. Training Testing and Validation

The proposed *LoCoMo-Net* framework was implemented on a Lenovo ThinkStation with an Intel Xeon CPU E5-2650 v2 processor @ 2.6 GHz and 32 GB RAM. The model is trained-tested and validated on Keras 2.2.0 environment configured to use Tensorflow 1.9.0 as backend engine on a 64 bit Ubuntu operating system. For real-time execution, we have prototyped the *LoCoMo-Net* model on Virtex-7 Xilinx FPGA platform and performed the validation.

### F. Proposed Two Stage Pipeline

#### 1) Proposed Input Data Compression Module

The input size is one of the parameters that impact the computational behavior of the CNN model; larger the size of the input data more would be the computational cost for the CNN model. To reduce the computational cost, we have developed a technique named ‘ *Min/Max’* to extract the relevant information from the sEMG signal. This was done by finding the local minima/maxima from the input signal which is based on the recent study by Phinyomark and Scheme [29] who investigated the use of local peaks (maxima) and valleys (minima) as a time domain feature for sEMG signal classification. The block level architecture is shown in Fig. 5(a) for the proposed ‘ *Min/Max’* was designed based on [36], [37] wherein a local maxima/minima are defined as a data sample which is not equal to (either larger or smaller) than the two neighboring samples. Two comparators simultaneously check whether $X_{i+1}$ is greater than the $X_{i+2}$ and $X_{i}$ and then result are passed to AND gate which enables a signal (ENB_$1= 1$) to store $X_{i+1}$ as local maxima. Similarly, if $X_{i+1}$ is smaller than $X_{i+2}$ and $X_{i}$, then the output of the two comparators are passed through a NAND gate which enables a signal (ENB_$2=1$) to store $X_{i+1}$ as local minima.

The effectiveness of the ‘ *Min/Max* ’ technique was analyzed with the mean entropy information of the sEMG signal data input of 250 samples and compressed to 146 samples for all the tasks and subjects of DS1 which is shown in Fig. 5(b). It is seen that the compressed data retain the most important information for all the able-bodied and amputated participants. Additionally, we performed the comparative analysis of software results for finding maxima/minima using ‘ *Findpeaks* ’ function available in MATLAB with our proposed ‘ *Min/Max’* technique which is shown in Fig. 5(c).

**FIGURE 6.**
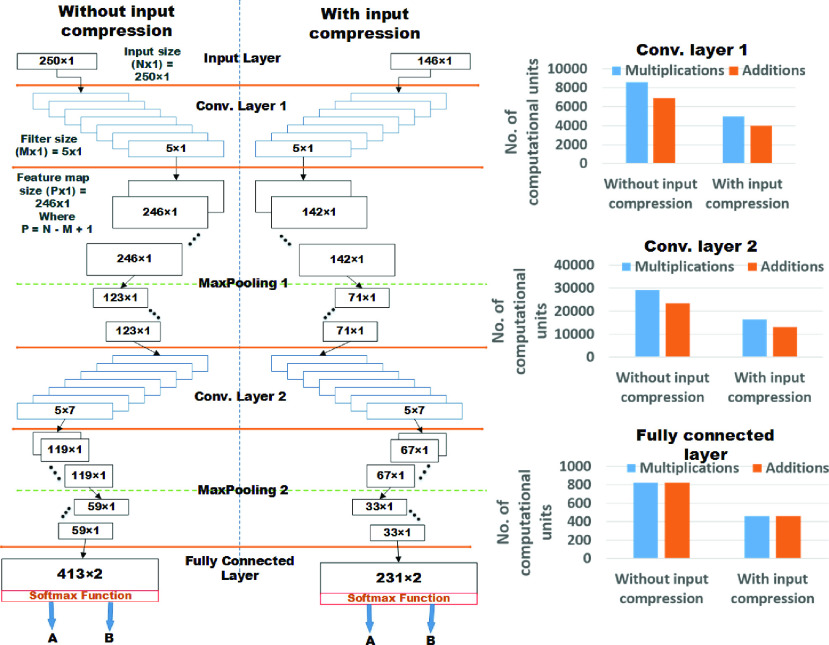
Shows influence of input compression on the computational cost of the model where for the calculation of total number of multiplication and addition units in a particular layer is done by considering $N$ kernels of size $F_{V}=F_{A}\times F_{B}$ is getting convolved with $K$ input feature map of size $I_{V} = I_{A}\times I_{B}$ with stride rate of $s$, which results in $N$ output feature maps of size $O_{V} =O_{A}\times O_{B}$. Thus, total number of multiplication ($M_{j}$) and addition ($A_{j}$) operations at $\text{j}^{\mathrm {th}}$ layer are $M_{j} = [(O_{V}\times F_{V})\times K] \times N$ and $A_{j} = [\{O_{V}\times (F_{V} - 1)\} \times K] \times N$ respectively.

The influence of the proposed *‘Min/Max’* technique on the architectural parameters of the proposed network topology is demonstrated in Fig. 6 wherein the left and right half portion of the figure depicts the architecture of the proposed network topology for uncompressed (250 samples) and compressed input (146 samples) respectively. The proposed ‘ *Min/Max’* has reduced the input data size by approximately 42% with negligible loss in accuracy. This reduced the computational cost of our proposed network topology which is detailed in the discussion section IV-A.

#### 2) Proposed Data-Driven Weight Sharing Module

**FIGURE 7.**
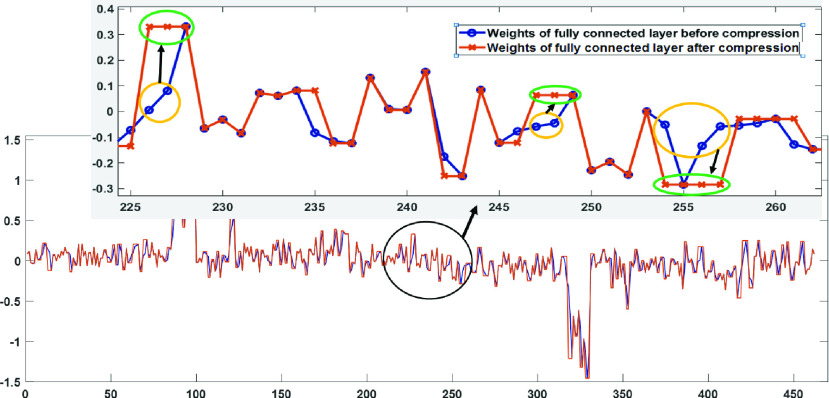
Shows an example of the data-driven weight sharing module. Where weights of the fully connected layer before compression and after compression (yellow color show the weights replaced with their corresponding local maxima or minima depicted with green color in the zoomed plot) are represented with blue and red color respectively.

In this module, we proposed a data-driven weight sharing technique for the compression of weights of the trained model to make a memory efficient design. The data-driven weight sharing module can inherently allocate the weight of maximum magnitude to all the neighboring weights with less magnitude present at an interval of time. As a result, multiple connections share the same weights. Thus, only the effective weight and the indices need to be stored. The weights of the fully connected layer when plotted resembles an asymmetrical triangular waveform and the essence of the proposed module is that at an interval of time the fewer magnitude weights are replaced with the more magnitude weight present among them. Thus, the output of the compressed weights when plotted look a lot like an irregular square and rectangular waveform based on the magnitude of weights as shown in Fig. 7. Since, the weight sharing is applied to the trained model weights, therefore it does not require real-time processing. This has been performed offline in MATLAB by loading the model file from python and the modified weights used for further analysis.

### G. LoCoMo-Net Hardware Architecture

**FIGURE 8.**
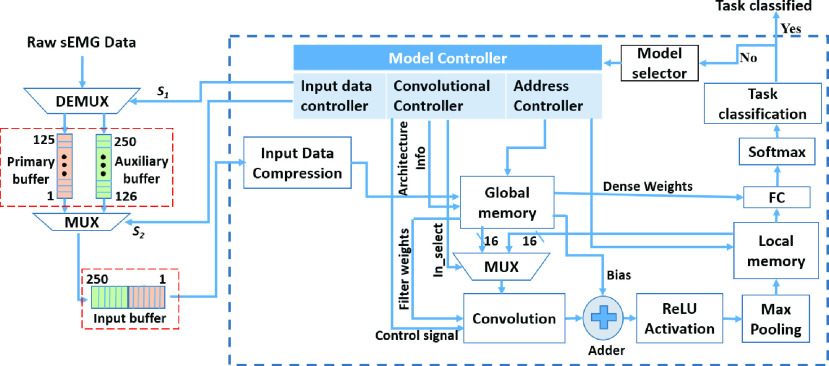
Block level architecture of the proposed LoCoMo-Net model.

The block level architecture of the proposed *LoCoMo-Net* for real-time implementation is illustrated in Fig. 8. It includes model controller, consisting of three parts: (i) Input data controller; (ii) convolutional controller; and (iii) addressing controller for monitoring and controlling the input data processing; architectural parameters (like filter size, input size, and filter depth); and flow of weight parameters respectively. The approach used was similar to reported in the literature [38] for sEMG data segmentation in real-time using a 250 ms window with 50 % overlap. The classification process starts with the 1st classifier where all the weights of the 1st classifier are enabled in the initial stage. Later, based on the output from the task classification module, further computation is performed. The steps for hardware implementation of the CNN model are as follows-

1) The input data compression compresses the sEMG signal from 250 samples to 146 samples, which are then stored on the global memory along with the weight parameters of 15 classifiers.
2) Control signal enables the convolution module to start performing the convolution between the filter and compressed sEMG data.
3) ReLU and max-pooling computation are pipelined with convolution operation to eliminate the storage for intermediate feature maps in convolution and ReLU stages. The output of pooling module is stored into the local memory.
4) The intermediate data from the local memory are sent to the convolutional module again to perform the convolution for the 2nd convolutional layer.
5) Next, ReLU and pooling for the 2nd convolutional layer are performed, and outputs are overwritten in the local memory.
6) Now, the intermediate data from the local memory are passed to the fully connected (FC) module where matrix multiplication is performed. This resulted in an output having two neurons which are sent to the softmax module for classification.
7) The softmax module assigns the ‘1’ and ‘0’ values to the neurons based on high and low probability respectively. If the output is ‘1’ (high), then it shows the input sEMG belongs to the 1st classifier, i.e., Task 1 is classified. Otherwise, next classifier’s weights are loaded, and the same process is repeated from stage 2 to 6 until the softmax module outputs ‘1’ (high).

### H. Performance Assessment

In this study, to validate the performance of the proposed *LoCoMo-Net* model, we have done a comparative analysis with different models using the same network topology which are as follows-

1. *Conventional No-compression (NC) model*: In this model, the CNN model parameters are identical to the *LoCoMo-Net* model except it doesn’t include the input data compression module and data-driven weights compression module.
2. *Proposed Input-compression (IC) model*: In this model, the CNN model parameters are identical to the *LoCoMo-Net* model except it includes input data compression module and excludes data driven weight sharing module.
3. *Proposed LoCoMo-Net model:* This proposed model, includes the input data compression module followed by the data-driven weight sharing module which resulted in reduced computational and memory demand compared with NC and IC models.

## III. Results

**FIGURE 9.**
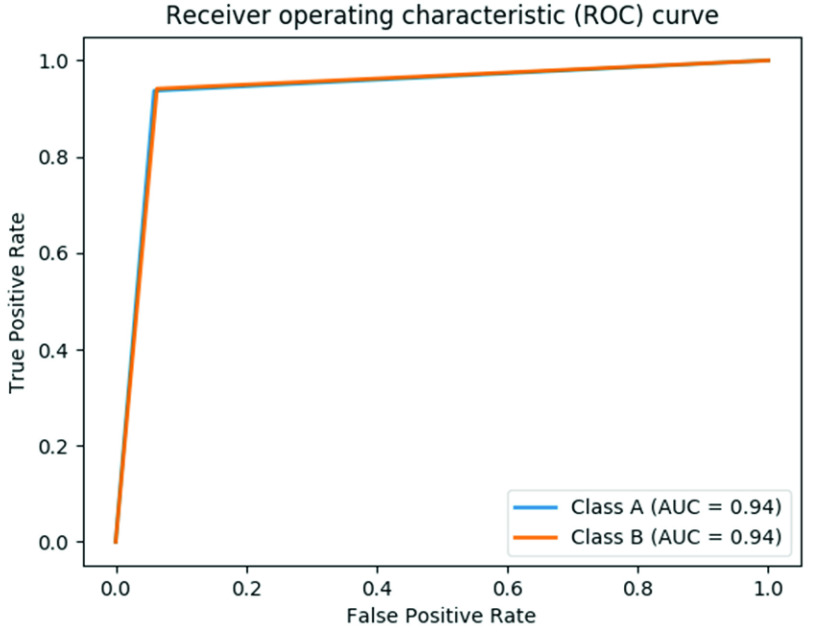
ROC curve of LoCoMo-Net model.

Table 1 and 2 show the comparative analysis of neural net architectures and performance metrics of NC, IC and *LoCoMo-Net* models respectively. The low-complexity architecture of the proposed *LoCoMo-Net* with negligible loss in accuracy compared to NC and IC validates the proposed two-stage pipeline technique. For classification, all 50 movements of DS2 were considered at the same time but for ease of understanding the average classification accuracy of basics movements, grasping and functional movement and force pattern were given individually in Table 2 (including able-bodied and amputated participants). The average classification accuracy achieved by *LoCoMo-Net* model for all movements of 11 able-bodied subjects, 3 amputated subjects, DB2 and DB3 is 93.4%, 88.8%, 94.7%, and 88.7% respectively. Precision, recall, and F1 score are considered effective metrics for imbalance class distribution therefore, we have included these metrics in our analysis for *LoCoMo-Net* model which are given in Table 2. The average precision, recall and F1-score for all movements of 11 able-bodied subjects: 93.8, 98.2 and 95.9; 3 amputated subjects: 92.3, 93.6 and 92.9; DB2: 95.4, 98.2 and 96.7; DB3: 91.6, 93.9 and 92.7. Further, most of the existing works performed the analysis with 200ms window length, therefore, we have also included the analysis with 200ms window length in Table 2 for fair comparison with the existing works. Furthermore, the receiver operating characteristics (ROC) curve was generated with a true positive rate (TPR) against the false positive rate (FPR) to measure the separability of classes for the LoCoMo-Net model. This is depicted in Fig. 9, where the area under the curve (AUC) is 0.94.

### A. Hardware Complexity Analysis

**FIGURE 10.**
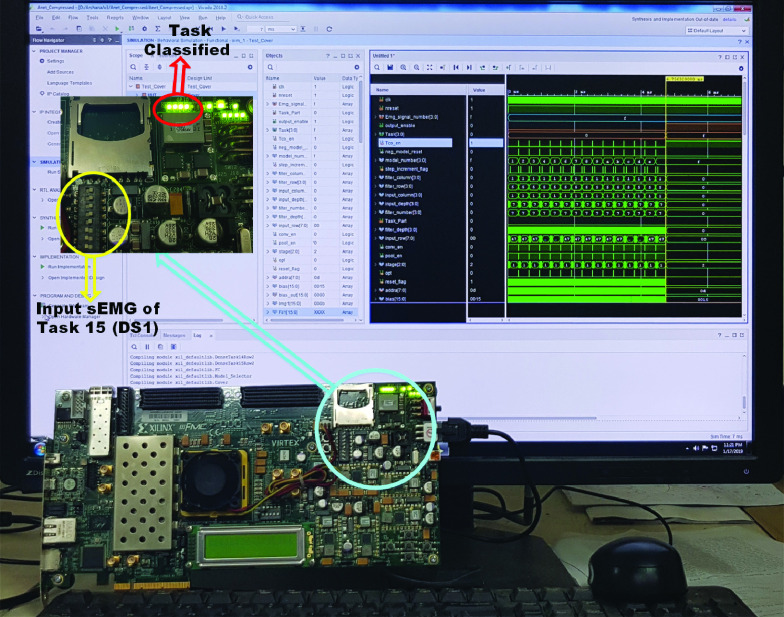
FPGA prototyping of the *LoCoMo-Net* model shown for Task 15 (DS1), where red color circle indicates output with illuminating LED and yellow color circle indicates input knob.

The proposed LoCoMo-Net was prototyped on the Virtex-7 Xilinx FPGA platform (Fig. 10) for a 16-bit word length with a clock frequency of 100 MHz for assessing its real-time capabilities. The NC and the proposed IC model were also implemented for comparative analysis of their computational complexity. The results show that the proposed *LoCoMo-Net* model utilizes fewer resources and consumes less power compared to conventional NC model which is tabulated in Table 3. The total computational time and power consumption of *LoCoMo-Net* model is approximately 4.7 ms and 137 mW respectively, showing the feasibility of proposed *LoCoMo-Net* in real-time settings.

The proposed model was formulated for binary task classification, therefore, the weights for each task need to be stored individually. However, only one hardware implementation for the classifier is required for all the tasks. Thus, 15 task weight parameters and one classification hardware were required for classifying 15 movements from DS1. The results reported in Table 3 shows the model complexity of the proposed LoCoMo-Net model for classifying 15 movements. Further, in the classification of 50 movements from DS2, the hardware complexity remains the same but the computational time increases due to serial execution. Thus, the proposed two-stage pipeline: the input data-compression followed by data-driven weight sharing resulted in reduced computational time by the extent of data compression.

### B. Benchmarking of the Proposed LoCoMo-Net Model

We have benchmarked our *LoCoMo-Net* model with the other work [15] which has considered these 15 movements from DS1. In comparison, research reported in this manuscript has investigated the performance on the data from 11 able-bodied and 3 trans-radial amputated participants while their work used recordings from 9 subjects: 8 able-bodied and 1 amputated participant. To ensure unbiased comparison, we have evaluated the performance based on the average accuracy of the able-bodied and amputated participants. The results are shown in Table 4, which show that the *LoCoMo-Net* has approximately 8.5% increase in classification accuracy compared to Twin-SVM for all the 15 movements for 250ms input window length.

One of the obvious strength of this study is that it performs unsupervised feature extraction and learning while earlier comparable studies [15] used one feature, i.e., root mean square (RMS). The traditional methods of sEMG based movement classification rely on handcrafted feature selection followed by extraction before classifying the intended movements as shown in Fig 1. As a result, this process put a significant amount of time due to the human intervention and exertions in finding suitable features, and feature extraction increases computational time and complexity. Therefore, with the pre-determined window size, data-driven feature selection and extraction leads to higher accuracy and shorter delays compared with traditional methods.

### C. Comparison With the State-of-the-Art CNN Models for sEMG Hand Movement Classification

There are very few studies presented in the literature on the CNN based sEMG hand movement classification [9], [19], [20], [21], [42] and [43]. A comparative summary of the architectural overview and classification accuracy of the state-of-the-art CNN models with the proposed *LoCoMo-Net* model are reported in Table 5. The study by [9], [19], [21], [42] and [43] evaluated their model on the publicly available Ninapro database, i.e. DS2. Therefore, the comparison with these studies would be fair in terms of accuracy. The comparison results show that the proposed model achieved an improvement of 4%, 55.4%, 18.9%, 13.8% and 30.7% in accuracy compared to [9], [19], [21], [42] and [43] respectively. The study by [20] has shown an increase in performance compared to the proposed *LoCoMo-Net* model by 4.4%. However, this cannot be a fair comparison owing to the fact that they have evaluated their model on diffeent dataset, having less number of movement types compared to the propsoed methodology [10]. The parameters of the deep-learning models such as input size, input type (1D or 3D), filter size, number of filters, convolution layers, pooling layers, and the fully connected layer directly contribute to the computational complexity of the model as given in Table 1 and Fig. 6. The comparison of state-of-the-art CNN models with the proposed model on computational complexity was done based on the input type, number of layers, filter size, number of filters given in Table 5. The model complexity of the proposed *LoCoMo-Net* model described in Table 5 is based on our hardware implementation where the same hardware will be reused (serial computation) for classifying 50 movements. Thus, it results in total computational time (worst case) of approximately 15.8 ms ($317\,\,\mu \text {s}\times 50 + 5\,\,\mu \text{s}$). The total time, the sum of input window length and computational time equals to 265.8 ms (250 ms +15.8 ms), which is within the acceptable limit of real-time prosthetic control, i.e., 300 ms as given in the literature [31]. In the case of parallel computation of the *LoCoMo-Net* model, the model complexity will be 50 times for 50 movement classification while maintaining the computation time to only $317~\mu \text{s}$. This shows the trade-off between computation time and model complexity. Further, all of these state-of-the-art models [9], [19], [21], [42], [43] have to pay the penalty in terms of latency and extra-hardware cost due to the requirement of the 3D spectrogram as an input. Therefore, all the models in the literature are computationally heavily expensive and would need huge memory for storage when compared to proposed *LoCoMo-Net* model.

## IV. Discussion

The performance of a single channel sEMG signal for real-time recognition of the user movements was achieved by compressing the data prior to CNN which reduced the computation cost. The input data compression was followed by data-driven weight sharing concept to make the proposed *LoCoMo-Net* less memory-intensive for execution on resource constrained platform. This study has also investigated the impact of number of channels and compression on the behavior of deep learning model which is detailed further in this section.

### A. Significance of Proposed Two-Stage Compression

*One Stage: Input data compression:* The influence of input size (number of samples) on the computational behavior, i.e., output shape and the total number of parameters on the same CNN architecture can be interpreted from Fig. 6 and Table 1. This shows the IC model requires less computation and resources compared to the NC model. The same has been tested by real-time prototyping on FPGA platform wherein IC model requires 30%, 37%, 41%, 17%, and 43% less number of LUTs, registers, memory, power consumption and computational time respectively in contrast to NC model given in Table 3. While time lost with compression of input data (*One Stage: Input data compression*) is $5~\mu \text{s}$, hence makes it feasible for execution in real-time.

The entropy based analysis is shown in Fig. 5(b) and the study reported by [29], confirmed that the proposed *Min/Max* technique extracts the relevant information present in the signal. Similarly, the comparison of the performance with IC and NC models show insignificant accuracy loss (Table 2). Thus, the input compression module or feature extraction [29] did not alter the performance but reduced the complexity, making it suitable for real-time implementation.

*Two Stage: Input data compression followed by data-driven weight sharing:* The performance of input data compression followed by data-driven weight sharing is given in Table 1, 2 and Table 3. The total number of different weights in the FC layer of NC, IC, and LoCoMo-Net model are 828, 464, and 294 respectively. This shows a reduction of approximately 65% and 26% in the number of weights of the proposed LoCoMo-Net model with an expense of approximately 0.35% and 0.1% accuracy loss compared to NC and IC model respectively. However, a similar impact was shown from the hardware implementation results presented in Table 3. The proposed LoCoMo-Net model required 27%, 49%, 50%, 23% and 43% less number of LUT’s, registers, memory, power consumption and computational time respectively compared to NC model.

### B. Scalability of the LoCoMo-Net Model

**FIGURE 11.**
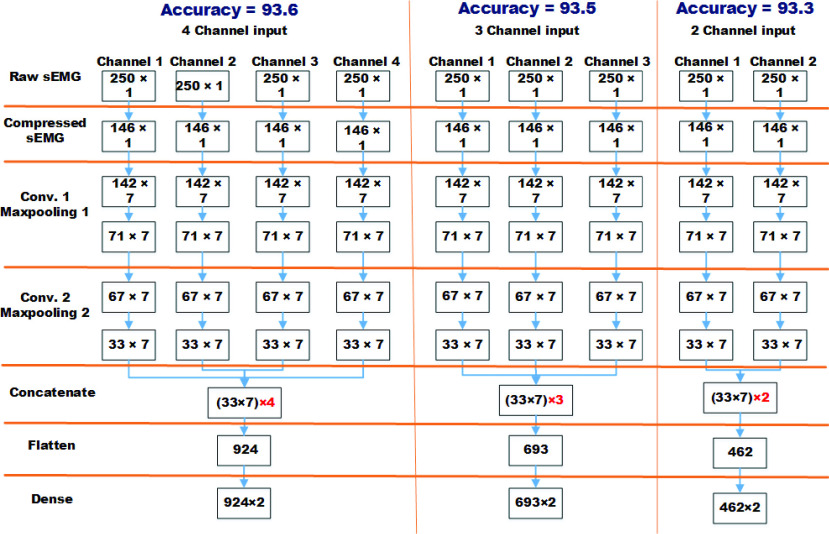
Scalability analysis for different number of channels.

The scalability of the proposed *LoCoMo-Net* model was tested for a number of channels ranging from 1 to 4. It was found that improvement in the accuracy with the increase in the number of channels was small. However, the model complexity increased by $n$ times for $n$ number of channels, as shown in Fig. 11. This makes the implementation for real-time application requiring $n$ parallel hardware, which increases the cost and complexity. The scalability of the *LoCoMo-Net* model with respect to $n$ number of channels can be represented by equation 1.

\begin{equation*} Model=n\times (C_{1}+C_{2}+P_{1}+P_{2})+F+D\tag{1}\end{equation*}

where $C_{1}$, $C_{2}$, $P_{1}$, $P_{2}$, $F$, and $D$ are *Conv1D_1*, *Conv1D_2*, *Pooling_1*, *Pooling_2*, *Flatten* and *Dense* layers respectively. The average classification accuracy of 11 able-bodied subjects (DS1) for 1 channel, 2 channels, 3 channels and 4 channels are 93.4%, 93.3%, 93.5% and 93.6% respectively. Therefore, to reduce the computational-complexity single channel sEMG is included in the proposed *LoCoMo-Net*.

## V. Conclusion

The proposed deep learning framework *LoCoMo-Net* based on CNN has two-stage pipeline compression: 1) input data compression and 2) data-driven weight sharing. This design reduces the number of LUTs, registers, memory, power consumption and computational time by 27%, 49%, 50%, 23% and 43% respectively compared to NC model without sacrificing the performance. In addition, the proposed *LoCoMo-Net* shows an average improvement in the performance of approximately 8.5% and 16.0% in contrast to Twin-SVM and the recent CNN model [21] respectively. The results from our investigation regarding performance enhancement as well as hardware efficiency can be considered favorable for the potential implementation in the real prosthetic limb applications.

## Appendix

See Tables 6 and 7.
